# 3D‐Printed Milli‐Fluidic Mixing‐Extrusion Platform for Continuous, High‐Throughput LNP Production

**DOI:** 10.1002/smll.74386

**Published:** 2026-07-03

**Authors:** Leekang Jeon, Seeun Kim, Hanjin Seo, Hyun Su Lee, Gi‐Su Na, Gi‐Ra Yi, Hyomin Lee

**Affiliations:** ^1^ Department of Chemical Engineering Pohang University of Science and Technology (POSTECH) Pohang Gyeongbuk South Korea

**Keywords:** 3D‐printing, extrusion, high‐throughput, lipid nanoparticles, mixing

## Abstract

Lipid nanoparticles (LNPs) have emerged as a prominent delivery vehicle for therapeutics such as mRNA vaccines and genetic medicines. However, current manufacturing technologies struggle to balance precision, scalability, and cost‐effectiveness. While microfluidic mixing offers excellent particle control, it often faces operational challenges, including channel fouling and a reliance on costly single‐use chips that create bottlenecks for reproducible and scalable production of LNPs. Conversely, bulk methods lack uniformity, and conventional extrusion remains a labor‐intensive batch process. Here, we report a hybrid continuous synthesis platform that integrates a reusable 3D‐printed milli‐fluidic mixer with inline membrane extrusion. By engineering a robust 3D internal architecture at the milli‐scale, this system achieves rapid mixing while mitigating clogging risks associated with micro‐channels. Our platform also offers a durable, easy‐to‐clean alternative that enhances process reproducibility while the integrated design enables single‐pass refinement of LNPs, streamlining the production workflow to achieve high‐quality, uniform LNPs at flow rates of up to 20 mL min^−1^. Overall, our study provides a scalable, cost‐effective, and contamination‐resistant pathway for industrial LNP manufacturing, overcoming the limitations of existing disposable cartridge‐based systems.

## Graphical abstract

We report a continuous high‐throughput production of lipid nanoparticles (LNPs) by integrating a reusable 3D‐printed milli‐fluidic split‐and‐recombine mixer with inline membrane extrusion. The 3D architecture enables rapid early‐stage mixing, reducing clogging and fouling, while inline extrusion further refines the as‐formed LNPs, improving uniformity, yield, and storage stability. This hybrid mixing‐extrusion workflow produces high‐quality, gene‐loaded LNPs up to 20 mL min^−1^.

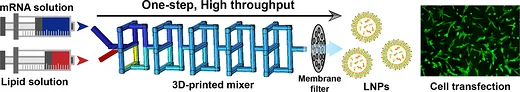

## 1 Introduction

Lipid nanoparticles (LNPs) have become one of the most widely adopted delivery platforms for small‐molecule therapeutics and nucleic acids [1, 2, 3, 4], and now represent a core enabling technology for mRNA vaccines [5, 6], gene editing [7, 8], delivery of diverse macromolecular payloads, and high‐throughput screening applications [9, 10, 11]. LNP synthesis typically involves the spontaneous self‐assembly of nucleic acids (e.g., DNA or RNA) with a lipid formulation containing ionizable lipids, PEG‐lipids, phospholipids, and cholesterol, dissolved in organic solvents such as ethanol. Upon mixing with an acidic aqueous buffer containing nucleic acids, the ensuing solvent dilution and protonation of ionizable lipids increase electrostatic attraction to the nucleic acids, driving the spontaneous formation of nanoparticles [2, 12, 13]. During this process, the mixing kinetics between the organic and aqueous phases critically govern the resulting particle size and dispersity; rapid mixing accelerates faster solvent dilution, thereby suppressing the formation of aggregates, and yielding smaller, more uniform LNPs [14, 15]. Given that carriers often need to be effectively small to traverse endothelial gaps in lymphatic vessels [16, 17, 18], achieving consistent LNP characteristics through effective mixing is critical for robust delivery efficiency.

While conventional bulk mixing approaches remain attractive due to its simplicity and scalability, they often suffer from nonuniform, poorly defined mixing fields, which lead to broad size distributions and low encapsulation efficiency [4, 19, 20]. To address these issues, microfluidic approaches such as baffle mixers [14], herringbone mixers [15, 18], Tesla‐type architectures [21], and 3‐D geometric designs have been explored [22]. Rapid mixing in confined channels improves size controllability and encapsulation efficiency while enabling scale‐up production through parallelization [20, 23]. However, practical deployment of microfluidic approaches frequently faces operational challenges resulting from high surface area‐to‐volume ratios and low channel dimensions that inevitably lead to fouling and clogging of LNP precursors, compromising long‐term flow stability and particle uniformity [4, 24]. Although surface modification strategies can mitigate contamination to some extent, they add fabrication complexity and often fail to ensure long‐term stability [25, 26, 27, 28]. It is also noteworthy that current reliance on costly single‐use microfluidic chips in several commercial systems creates an economic bottleneck which limits their scalability and sustainability [4, 29].

Alternatively, membrane extrusion has been employed as a post‐processing technique to refine LNP size by passing bulk pre‐mix solutions through porous membranes [30]. In particular, ethanol content and pH are tuned during bulk mixing to maintain appropriate membrane fluidity, followed by extrusion to narrow the size distribution. While this method allows scalable production of uniform LNPs without complex equipment, it remains a discontinuous process requiring repeated steps. Moreover, maintaining the quality and stability of the pre‐mix solution is essential to ensure reliable LNP synthesis outcomes, complicating process control and increasing susceptibility to environmental perturbations such as temperature and pH fluctuations during scale‐up. Collectively, these approaches reveal a clear trade‐off among precision, scalability, and robustness, as bulk mixing scales well but lacks precision, microfluidics delivers precision but is operationally fragile and costly in consumables, and membrane extrusion improves size control while adding batch processing complexity. Consequently, there is still an unmet need for a unified platform that not only combines the precision of microfluidics with the scalability of bulk processes but also overcomes the economic and environmental limitations of cost‐effectiveness and reproducibility [31, 32, 33].

**FIGURE 1:**
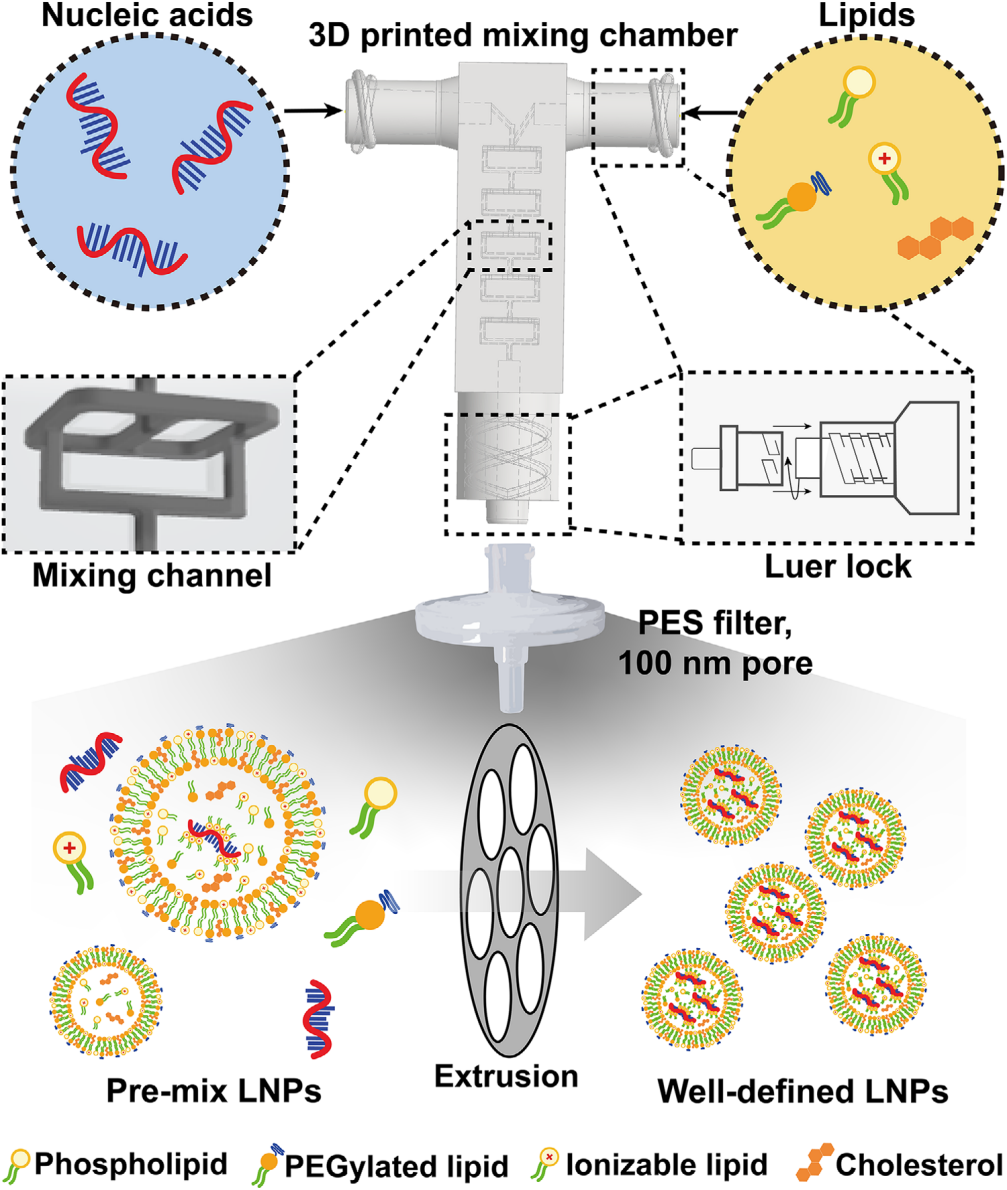
Schematic illustration of the hybrid mixing‐extrusion platform for LNPs production.

Herein, we present a hybrid continuous LNP synthesis platform that integrates a reusable 3D‐printed milli‐fluidic mixing chamber with inline membrane extrusion. The lipid and nucleic acid solutions are separately introduced into the inlets of the 3D‐printed mixing chamber, where the internal channel architecture promotes rapid mixing to generate a pre‐mixed LNP suspension as depicted in Figure 1. The outlet stream is subsequently routed through an inline extrusion filter to produce uniform, well‐defined LNPs, and the workflow remains mechanically robust under high flow rates and elevated pressures. An overall view of the 3D mixing chamber assembled with the filter and syringes is shown in Figure S1, and a video of the LNP synthesis process is provided in Movie S1. By simultaneously expanding the channel dimensions from micro‐ to milli‐scale and engineering a robust 3D internal mixing architecture, our system successfully achieves high mixing efficiency at elevated flow rates while mitigating susceptibility to clogging and surface fouling. Unlike conventional passive mixers that rely on localized interfacial perturbation within a single plane, our 3D architecture forces the entire fluid stream to repeatedly split and recombine across alternating planes. In addition, operation in milli‐scale channels (500 µm) at high flow rates places the system in an inertial‐dominated regime (Reynolds number ≈1,333), where global multi‐directional redirection enhances interfacial deformation and accelerates mixing beyond the diffusion‐limited behavior typical of larger channels. Moreover, our 3D‐printed mixer is durable and easy to clean, enabling repeated use without performance loss while improving reproducibility and reducing reliance on disposable systems. The resulting uniform and stable LNP pre‐mix is immediately processed through a membrane filter in a single pass, eliminating repeated extrusion cycles. Using this integrated platform, we demonstrate continuous, modular, and contamination‐resistant production of near‐100 nm LNPs at flow rates up to 20 mL min^−1^. We believe that this study represents a highly reproducible and scalable route toward multi‐liter‐per‐hour manufacturing of high‐quality LNP formulations, while remaining cost‐effective.

## 2 Results and Discussion

### 2.1 Effect of Mixer Architecture on Mixing Efficiency and LNP Size

**FIGURE 2:**
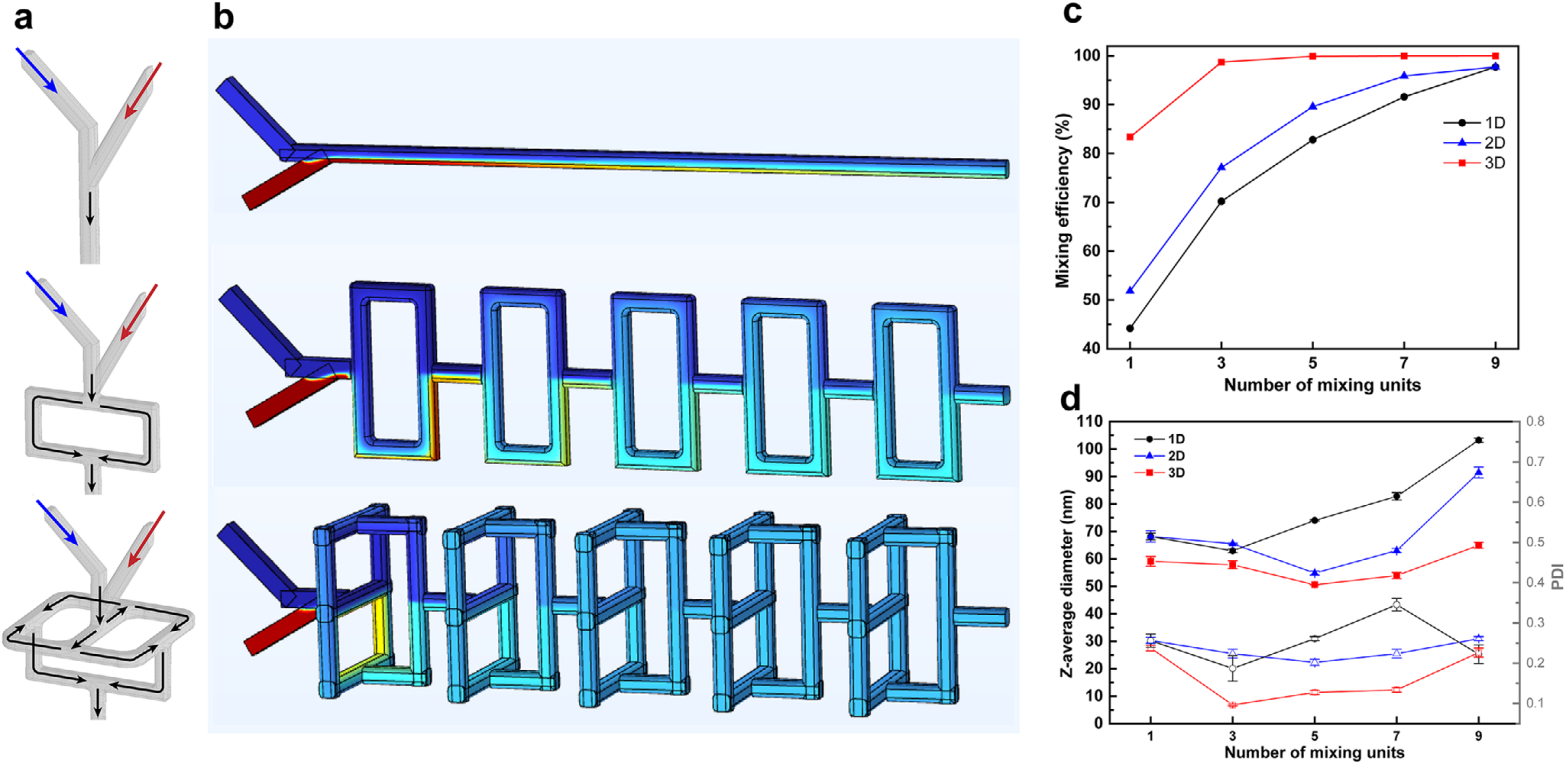
Effect of channel geometry on mixing efficiency and LNP size. (a) Schematic illustration of fluid flow in 1D, 2D, and 3D mixers, and (b) numerical simulation of fluid mixing using COMSOL Multiphysics for visualization. Water and ethanol are represented in blue and red, respectively. (c) Simulated mixing efficiency versus the number of mixing units for 1D, 2D, and 3D mixer architectures. (d) LNP size distributions and batch‐to‐batch variability for nanoparticles produced using 3D‐printed 1D, 2D, and 3D mixers. Black circles, blue triangles, and red squares indicate 1D, 2D, and 3D mixer condition, respectively. Closed symbols denote the Z‐average diameter, whereas open symbols denote the polydispersity index (PDI). Error bars in (d) represent the standard deviation (SD), where the values are reported as mean ± SD (*n* = 3).

To first evaluate the performance of the 3D‐printed milli‐fluidic mixing chamber as a function of mixer architecture, we designed three channel geometries denoted 1D, 2D, and 3D, as illustrated in Figure 2a, and performed numerical simulations in COMSOL Multiphysics and synthesized LNPs using corresponding 3D‐printed mixers. The 1D channel comprised a straight mixing segment without internal features, while the 2D channel incorporated a planar split‐and‐recombine motif. In the 2D channel, the flow was divided into two branches, routed through a rectangular loop within a single plane, and recombined downstream. In the 3D channel, the stream underwent a two‐step split‐and‐recombine process, in which the flow was first bifurcated and subsequently split orthogonally to generate four sub‐streams, followed by sequential recombination into a single downstream. We then simulated water‐ethanol concentration fields for the 1D, 2D, and 3D channels containing five mixing units, as shown in Figure 2b and Movie S2. The 1D channel exhibited predominantly laminar co‐flow with minimal interfacial deformation, such that intermixing occurred primarily by diffusion across the interface. In the 2D channel, despite repeated splitting and recombination, the two streams remained largely segregated, indicating that collision‐driven interpenetration was limited and that diffusion remained the dominant transport mechanism [34]. In contrast, the 3D channel yielded substantially accelerated homogenization relative to the 1D and 2D analogs, which is attributable to repeated splitting and recombination combined with changes in flow direction that enhance interfacial stretching and folding. Next, we calculated mixing efficiencies as a function of number of mixing units in each architecture, as shown in Figure 2c. For the 1D and 2D channels, mixing efficiency gradually increased with the number of units. By comparison, the 3D channel exceeded 80% mixing efficiency after a single unit and reached >99.9% after five units. Considering that LNP formation is particularly sensitive to the early‐stage mixing conditions immediately after the lipid and nucleic acid streams come into contact, we anticipated the 3D architecture to yield improved LNP quality relative to the 1D and 2D designs.

To experimentally validate these predictions, the simulated designs were fabricated via 3D printing and used to produce yeast RNA‐loaded LNPs comprising phospholipid, PEGylated lipid, ionizable lipid, and cholesterol, followed by size characterization prior to dialysis (Figure 2d). Across all tested unit numbers, the 3D mixer consistently produced smaller particles and lower polydispersity index (PDI) than the 1D and 2D mixers. The 1D mixer showed an increase in LNP diameter with increasing unit number, whereas the 2D and 3D mixers exhibited a modest size reduction up to five units, followed by a gradual increase at higher unit counts. We note that the discrepancy between simulated and measured trends is likely due to differences in the effective mixing length sampled in each case. Specifically, simulations quantified mixing immediately at the outlet of each unit, but experimentally, the flow experiences additional transport during injection from the syringes to the mixer as well as jetting during the initial collection process. Consequently, especially at low unit numbers, the experimental system may have undergone more downstream mixing than captured by the unit‐exit simulation metric. Moreover, the degradation in LNP quality observed at higher unit numbers is consistent with a reduced initial mixing rate caused by pressure drop along longer channels, reinforcing that LNP size is governed primarily by early‐stage mixing performance rather than by the final, overall mixing efficiency. Based on these results, we selected the 3D mixer with five mixing units as the optimal configuration and used this design for all subsequent experiments.

### 2.2 Comparison of LNP Size and Stability Across Various Workflows

**FIGURE 3:**
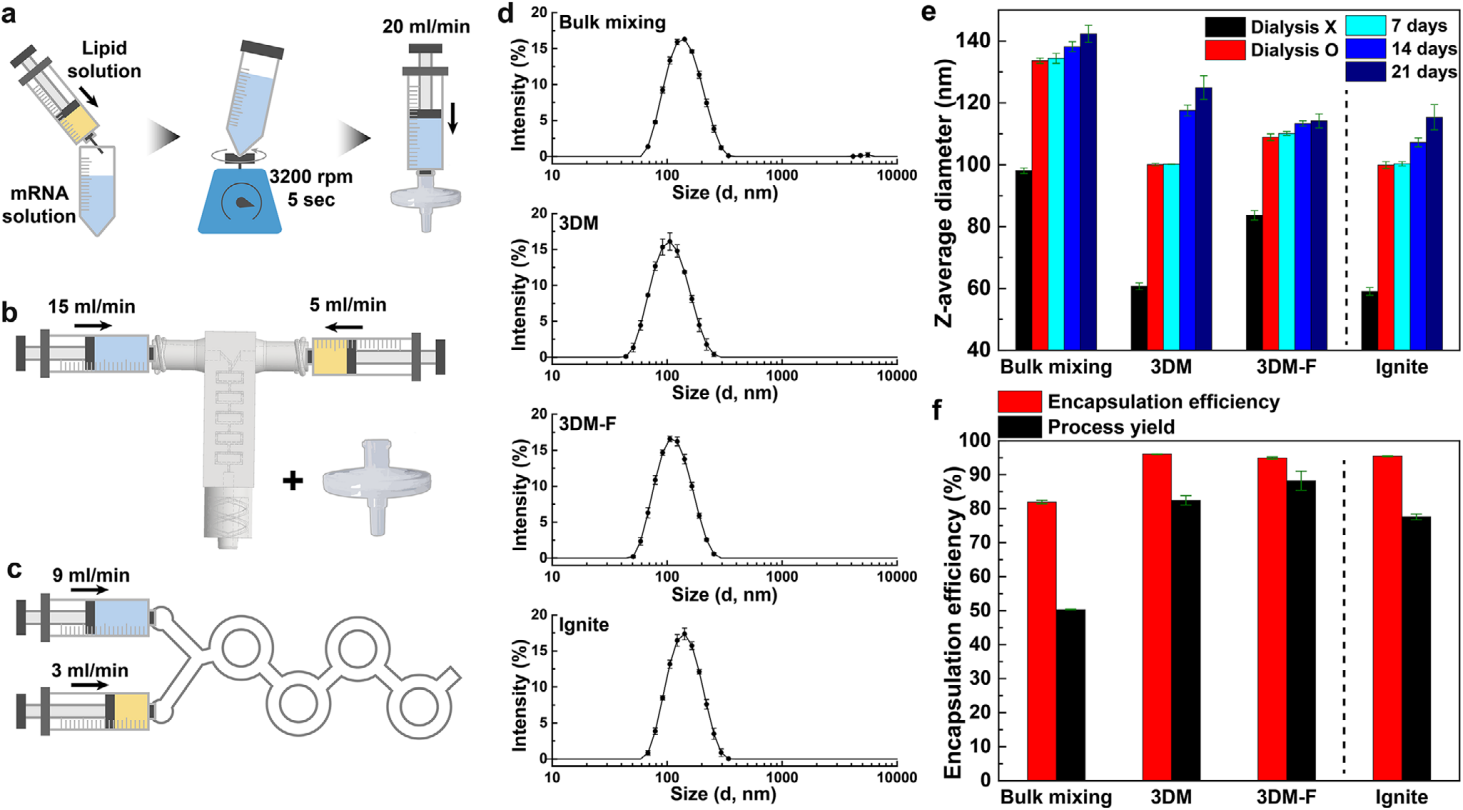
Characterization of LNPs produced using different synthesis workflows. (a–c) Schematic illustrations of bulk mixing using a vortex mixer followed by extrusion through an inline filter (Bulk mixing), the 3D‐printed mixer without (3DM) and with an inline filter (3DM‐F), and a commercial microfluidic mixer (Ignite, NanoAssemblr, Cytiva). (d) Particle size distributions of LNPs obtained from the four workflows after dialysis. (e) Comparison of hydrodynamic diameters measured before dialysis, after dialysis, and during weekly storage at 4°C. (f) Encapsulation efficiency and process yield of LNPs produced by each workflow. All error bars represent the standard deviation in which values are reported as mean ± SD (*n* = 3).

To examine the effect of fabrication method on LNP properties, we prepared sets of eGFP mRNA‐loaded LNPs using four representative workflows: (i) bulk mixing, in which the mRNA and lipid solutions were combined in bulk using a vortex mixer and subsequently extruded through an inline filter (bulk mixing, Figure 3a); (ii) our 3D‐printed mixing chamber operated either without or with an inline extrusion filter (3DM and 3DM‐F, Figure 3b); and (iii) a commercial microfluidic platform based on a ring‐type mixer (Ignite, Figure 3c). DLS measurement results acquired immediately after dialysis showed that among the four workflows, bulk mixing produced LNPs with a markedly larger Z‐average diameter (∼133.6 nm), while 3DM and Ignite yielded similar sizes (∼100.1 and 99.94 nm, respectively), and 3DM‐F produced slightly larger LNPs (∼108.9 nm) (Figure 3d). Next, we measured the temporal evolution of LNP size before and after dialysis, during three weeks of storage at 4°C, as shown in Figure 3e. For bulk mixing, limited initial mixing led to a highly heterogeneous pre‐mix with large particle populations prior to extrusion (Figure S2). Notably, even after passage through a 100 nm pore‐size filter, bulk‐mixed samples retained substantially larger diameters than the other conditions both before and after dialysis, indicating that the quality of the LNP pre‐mix upstream of filtration remains a critical determinant of final particle size. LNPs produced by 3DM and Ignite exhibited comparable sizes, whereas 3DM‐F yielded larger diameters prior to dialysis. After dialysis, the 3DM and Ignite samples showed a more pronounced size increase, reducing the size gap across conditions. As dialysis‐associated size growth has been attributed to fusion driven by an increase in external pH [35], we infer that passage through the filter is not simply a size‐exclusion step for removing larger particles but may instead promote shear‐driven nanoparticle reorganization during high‐pressure transit through the membrane. Consistent with this interpretation, shear‐induced particle reassembly has been reported in LNP processing methods such as extrusion and microfluidization [29, 36]. Upon storage at 4°C with weekly monitoring, LNPs prepared by 3DM‐F remained comparatively stable, whereas 3DM and Ignite exhibited a pronounced size increase after two weeks. Collectively, these trends support the notion that extrusion through a filter promotes particle reorganization and thereby improves colloidal stability, consistent with prior reports that incorporating a filtration step can suppress time‐dependent particle growth [37]. Notably, although all synthesized LNP formulations exhibited near‐neutral surface charge (within ±10 mV), 3DM‐F showed the largest absolute zeta potential among the tested samples, which is consistent with its improved colloidal stability (Figure S3).

Next, encapsulation efficiency and process yield were quantified using a RiboGreen assay (Figure 3f). Encapsulation efficiencies exceeding 90% were observed for the 3DM, 3DM‐F, and Ignite samples. In terms of process yield, 3DM‐F exhibited a modest improvement relative to 3DM and Ignite consistent with the hypothesis that forcing LNPs through a filter under elevated pressure facilitates particle reorganization and improves material recovery. In contrast, bulk mixing yielded lower encapsulation efficiency and process yield, likely due to the poor quality and heterogeneity of the initial pre‐mix. Taken together, these results indicate that generating a well‐controlled, small‐sized pre‐mix through efficient mixing, coupled with a subsequent extrusion‐driven reorganization step, is necessary for robust production of high‐quality LNPs.

### 2.3 In Vitro Cellular Assays of LNPs Produced from Various Workflows

**FIGURE 4:**
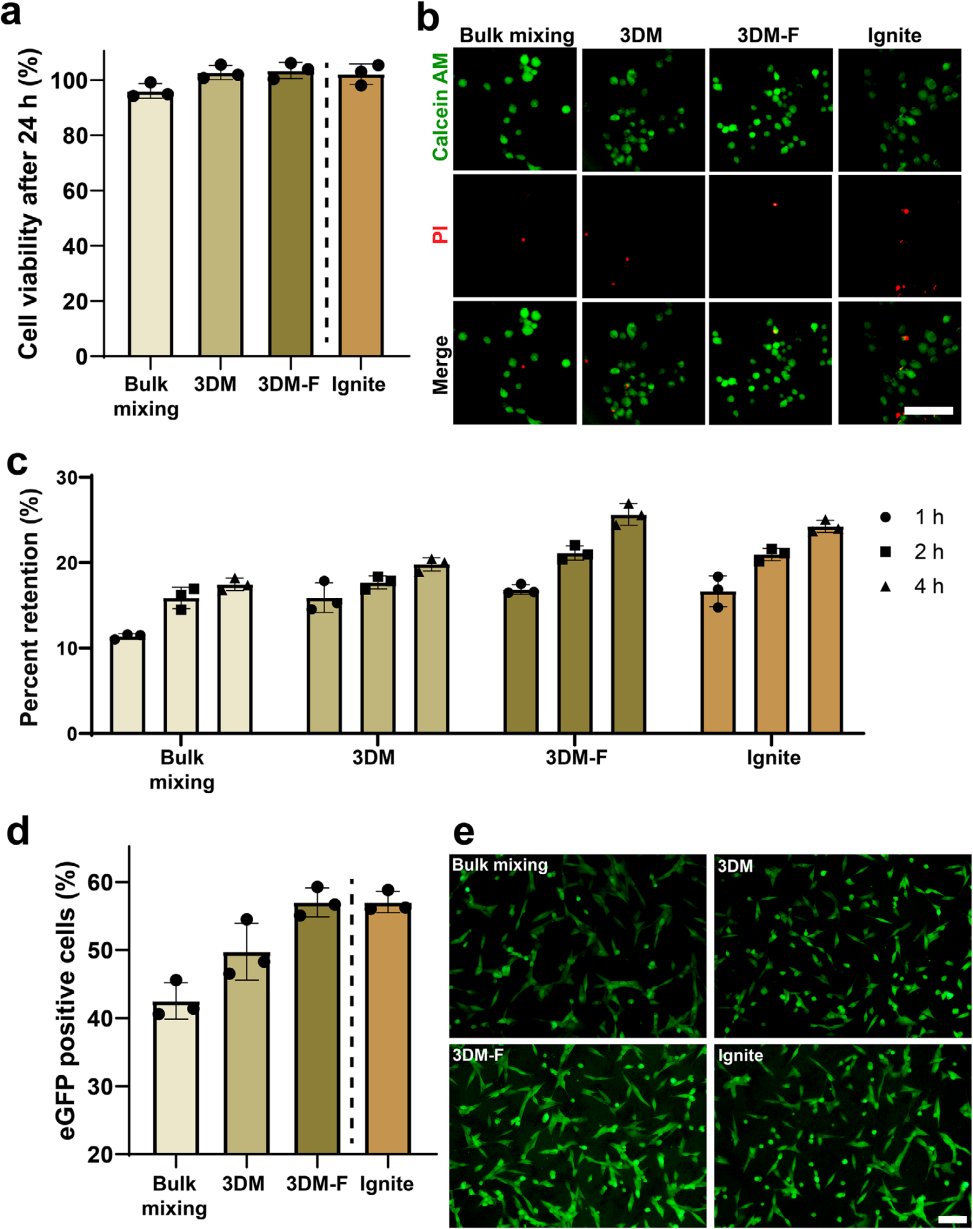
In vitro cellular assays of LNPs synthesized using four different workflows. (a,b) Cell viability following treatment with LNPs produced using four different synthesis workflows. Cell viability at 24 h was quantified using CCK‐8 assay, and live/dead staining was visualized using fluorescence microscopy. (c) Cellular uptake of fluorescently labeled eGFP mRNA‐LNPs. (d,e) In vitro transfection efficiency of LNPs and representative fluorescence microscopy images. All error bars represent the standard deviation, where the values are reported as mean ± SD (*n* = 3). All scale bars represent 100 µm.

To evaluate the biocompatibility and functional performance of LNPs produced using four different workflows, we conducted in vitro cell‐based assays using NIH‐3T3 fibroblasts. Cell viability was assessed by the CCK‐8 assay (Figure 4a) and by live/dead staining using calcein acetoxymethyl ester (Calcein AM) and propidium iodide (PI) (Figure 4b). All LNP formulations exhibited excellent cytocompatibility, maintaining >95% viability at 24 h post‐treatment, with similar high viability at 48 h (Figure S4). These results were consistent with live/dead imaging, which showed predominant green fluorescence (live cells) and minimal red signal (dead cells), indicating negligible cytotoxicity across formulations (Figure 4b). Cellular association of DiD‐labeled eGFP mRNA‐LNPs was also quantified after 1, 2, and 4 h of incubation (Figure 4c). All groups displayed time‐dependent increases in cellular association, and LNPs produced using the 3D‐printed mixing chamber with inline filtration (3DM‐F) showed an association profile comparable to the commercial Ignite formulation. To determine whether differences in cellular association translated into functional delivery, we evaluated eGFP expression 24 h post‐treatment (Figure 4d,e). LNPs were prepared at a dose of 1 µg mRNA per 1 × 10^5^ cells, and transfection efficiencies differed across workflows. Consistent with the cellular association trends, 3DM‐F LNPs yielded a higher fraction of eGFP‐positive cells (57.02 ± 1.73%) than bulk mixing (42.54 ± 2.19%) and 3DM (49.77 ± 3.43%) (Figure 4d). Fluorescence microscopy images further confirmed robust eGFP expression for the filtered 3D‐printed workflow, reaching levels comparable to the Ignite control (Figure 4e). Overall, these data demonstrate that LNPs synthesized using the 3D‐printed mixing chamber are highly biocompatible and that incorporation of an inline extrusion filter improves cellular association and transfection efficiency to a level comparable with an industry‐standard commercial microfluidic platform.

### 2.4 Long‐Term Operational Stability of the 3D Mixing Chamber and Cargo Versatility

**FIGURE 5:**
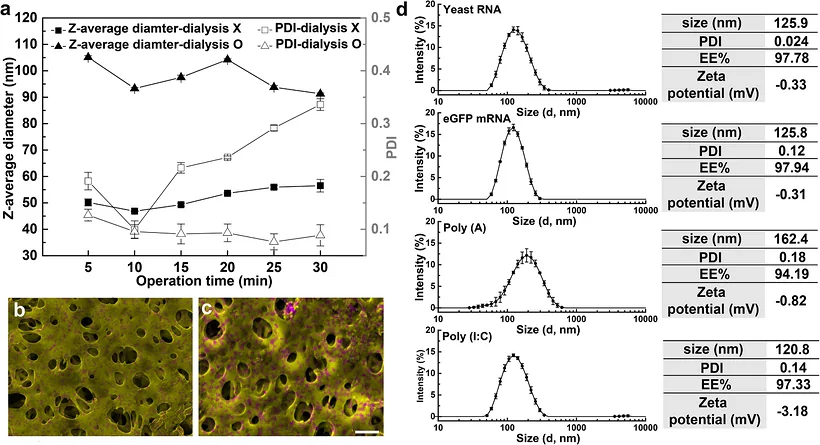
LNP size stability during extended operation of the 3D mixing chamber and fabrication of cargo‐variant LNPs. (a) LNP hydrodynamic diameter measured at 5 min intervals over a 30 min continuous synthesis run using the 3D mixing chamber. (b,c) Energy‐dispersive X‐ray spectroscopy (EDXS) images of the inline filter before and after LNP synthesis. Scale bar represents 1 µm. (d) Representative size distributions of yeast RNA‐, eGFP mRNA‐, poly(A)‐, and poly(I:C)‐loaded LNPs produced using the optimized 3D mixing chamber (3DM‐F). All error bars represent the standard deviation with values reported as mean ± SD (*n* = 3).

To evaluate the stability of the fabricated 3D mixer during continuous operation, yeast RNA (yRNA)‐encapsulated LNPs were produced at a flow rate of 20 mL min^−1^ for 30 min using the 3D mixer coupled to an inline filter (3DM‐F). Particle sizes collected at 5 min intervals and measured prior to dialysis are shown in Figure 5a. Throughout the 30 min run, the system consistently generated LNPs with diameters of approximately 50–55 nm. Although the PDI increased slightly over time before dialysis, it stabilized after dialysis, demonstrating that the platform can sustain the production of LNPs with diameters below 100 nm and PDI values below 0.1 over an extended period, as further supported by DLS analysis and cryo‐TEM imaging (Figure S5). Moreover, the use of an HPLC pump in place of a syringe pump further supports the scalability of the system toward fully continuous operation (Figure S6). As the larger channel dimensions of the 3D mixer are expected to reduce clogging susceptibility associated with fouling compared with chip‐based microfluidic mixers, we analyzed the surface of the used filters by energy‐dispersive X‐ray spectroscopy (EDXS) as presented in Figure 5b,c showing images acquired before and after the 30 min synthesis run, with sulfur (S K‐series) and phosphorus (P K‐series) signals highlighted in yellow and pink, respectively. Trace nucleic‐acid‐associated residues were detected on the filter surface after operation; however, no evidence of pore blockage by LNP components was observed. To evaluate the reusability of the 3D mixer, the same 3DM‐F was operated for five consecutive runs, each for 30 s with a rinsing step in between using DI water, ethanol, and PBS (Figure S7). Even after five cycles of washing and reuse, the size and PDI of the synthesized LNPs remained highly uniform. Together, these results indicate that the integrated 3D mixer‐filter configuration is largely resistant to fouling‐related contamination, performance drift, and clogging during extended operation. Further optimization of the overall process configuration may enable longer‐duration, high‐yield production without frequent replacement of the mixer or filter.

Next, we formulated LNPs encapsulating multiple nucleic acids, including yRNA, eGFP mRNA, poly(A), and poly(I:C) to assess cargo versatility (Figure 5d). Here, poly(A) (polyadenylic acid) and poly(I:C) (polyinosinic‐polycytidylic acid) were also selected as model cargoes to evaluate the encapsulation of structurally distinct nucleic acids within LNPs. Poly(A) is a single‐stranded RNA (ssRNA) homopolymer of adenine nucleotides, whereas poly(I:C) is a synthetic double‐stranded RNA (dsRNA) comprising a poly(I) strand annealed to a poly(C) strand and is widely used as a viral dsRNA mimic. Across cargo types, the resulting LNPs exhibited consistently high encapsulation efficiencies (>90%) and near‐neutral zeta potentials. These findings suggest that the 3D mixer‐filter strategy provides a broadly applicable route for scalable LNP production using diverse nucleic acid cargoes. Nevertheless, formulation‐specific optimization may be required for certain payloads, as exemplified by poly(A)‐loaded LNPs, which displayed a larger mean diameter (∼162 nm) relative to the other cargoes (∼120 nm). Because nucleic acids differ in physicochemical properties that can influence nanoparticle internal organization [38], these effects can likely be mitigated by tuning formulation parameters (e.g., N/P ratio and lipid composition) and process parameters (e.g., flow‐rate ratio, as well as filter pore size and membrane material). We also note that replacing the original inline filter with a polytetrafluoroethylene (PTFE) membrane increased particle diameters despite the same nominal pore size (Figure S8). This observation suggests that filter materials, and potentially the surface properties of the 3D‐printed chamber, may also influence LNP formation and/or post‐mixing reorganization. A systematic investigation of material‐dependent membrane effects represents an important future direction for optimizing continuous post‐processing and maximizing the performance of high‐throughput LNP manufacturing platforms. Overall, our results support the utility of the 3D mixer‐filter platform as a robust and scalable workflow for producing LNPs with diverse nucleic acid cargoes.

## 3 Conclusions

In this study, we established a scalable, chip‐free manufacturing strategy for LNP synthesis by integrating a 3D‐printed milli‐fluidic mixer with continuous inline extrusion. Numerical simulations and experimental validation demonstrated that a 3D split‐and‐recombine architecture dramatically accelerates early‐stage mixing relative to 1D and 2D designs, enabling the formation of smaller, less polydisperse LNPs and identified the five‐unit 3D mixer as the optimal configuration. Benchmarking against conventional workflows revealed that manual bulk mixing produces heterogeneous, oversized pre‐mixes that cannot be fully refined by downstream filtration, whereas 3D‐printed mixing without filtration (3DM) achieves particle sizes comparable to a commercial microfluidic platform (Ignite) but shows reduced long‐term size stability. Importantly, incorporating inline filtration (3DM‐F) improved process yield and storage stability while maintaining high encapsulation efficiency (>90%), and this advantage translated into superior in vitro performance. We showed that 3DM‐F LNPs exhibited excellent cytocompatibility and higher cellular association and transfection efficiency than bulk mixing and 3DM, with performance comparable to the commercial microfluidic mixer. Furthermore, continuous operation experiments demonstrated consistent production of 50–80 nm LNPs with low PDI, minimal evidence of filter pore obstruction, and robust encapsulation across diverse nucleic acid cargoes, supporting operational durability and payload versatility. Accordingly, these results indicate that high‐quality LNP production requires both efficient initial mixing to generate a well‐controlled pre‐mix and a subsequent shear‐mediated reorganization step during filtration. Overall, we envision that 3D‐printed mixer‐based architectures provide a promising strategy for economical, scalable LNP manufacturing with modular adaptability for application‐specific formulation optimization toward biomedical applications.

## 4 Experimental Section

### 4.1 Materials

Ethyl alcohol (EtOH), cholesterol, citric acid, sodium citrate dihydrate, ribonucleic acid from torula yeast (yeast RNA) and Dulbecco's Phosphate Buffered Saline (DPBS) were purchased from Sigma–Aldrich. 1,2‐distearoyl‐sn‐glycero‐3‐phosphocholine (DSPC), 1,2‐Dimyristoyl‐rac‐glycero‐3‐methoxypolyethylene glycol‐2000 (DMG‐PEG2000) were purchased from Avanti Polar Lipids, Inc. Heptadecan‐9‐yl‐8‐((2‐hydroxyethyl)(6‐oxO‐6‐(undecyloxy)hexyl)amino)octanoate (SM‐102) was purchased from Cayman Chemical. ARCA eGFP mRNA was purchased from APExBIO. Polyadenylic acid (Poly(A)) was purchased from Roche. Polyinosine‐polycytidylic acid (Poly(I:C)) was purchased from InvivoGen. Dulbecco's Modified Eagle Medium (DMEM), fetal bovine serum (FBS), and Penicillin‐Streptomycin (Pen Strep), and 1,1′‐dioctadecyl‐3,3,3′,3′‐tetramethylindodicarbocyanine (DiD) were purchased from Thermo Fisher Scientific Inc. 3D printer resin (poly (methyl methacrylate) (PMMA)‐based 3DK HR1 micro 150 resin) was purchased from Carima Inc. Polyethersulfone syringe filters (PES, 0.1 µm, 16553‐k 0.1) were purchased from Sartorius Stedim Biotech GmbH. Polytetrafluoroethylene syringe filters (PTFE, 0.1 µm, SLVVR33RS) were purchased from Merck Millipore Ltd.

### 4.2 Image Acquisition

All fluorescence microscope images were acquired using an inverted microscope (Eclipse T*i*2, Nikon) equipped with a CMOS camera. (Zyla 5.5, Andor). Surface of the filters before and after LNP synthesis was examined using a field‐emission scanning electron microscope (FE‐SEM, JSM‐7800F Prime, JEOL).

### 4.3 Fabrication of 3D Printed Mixing Chamber

The channel network was designed with a square cross‐section (500 µm × 500 µm) with rounded corners (25 µm fillet radius). The inlet channels for the lipid and nucleic acid solutions were oriented at 30° and 45° relative to the main channel, respectively. To mitigate backflow during injection, a 700 µm step was incorporated at the junction leading into the mixing region. This geometry yielded an offset Y‐junction design in which the aqueous nucleic acid stream enters and contacts the main mixing channel first, followed by downstream introduction of the lipid stream. The inlet and outlet ports were configured with Luer‐lock female and male fittings, respectively. Devices were fabricated using a DLP‐based 3D printer (IMC 57; Carima Inc.) with a nominal XY resolution of 57 µm.

### 4.4 Numerical Simulation

Numerical simulations were performed using COMSOL Multiphysics (v6.1; COMSOL Inc.) for the three‐dimensional model of the mixer. Ethanol and water were modeled as incompressible Newtonian fluids, and the steady‐state velocity field was solved under laminar‐flow conditions using the Navier–Stokes equation. No‐slip boundary conditions were applied at all channel walls. Mixing was assessed by coupling the flow solution to a passive scalar transport model using the Transport of Diluted Species interface. The scalar concentration was prescribed as 4 mol m^−3^ at the water inlet and 0 mol m^−3^ at the ethanol inlet, and was allowed to undergo convection and diffusion throughout the computational domain with a diffusion coefficient of 1.0 × 10^−^^9^ m^2^ s^−1^. Each inlet channel had a square cross‐section of 0.5 mm × 0.5 mm. The computational mesh consisted primarily of tetrahedral elements with boundary layer refinement near channel walls. Volumetric flow rates were set to 15 mL min^−1^ for the aqueous phase and 5 mL min^−1^ for the ethanol phase, corresponding to average inlet velocities of 1.0 and 0.333 m s^−1^, respectively, based on the inlet cross‐sectional area. Mixing efficiency was quantified from the standard deviation (σ) of the scalar concentration sampled at 400 points across the cross‐section of the merged downstream channel after each mixing unit. The reference standard deviation for the unmixed state (σ_i_) was calculated from an idealized two‐stream distribution consisting of 100 points at 0 mol m^−3^ and 300 points at 4 mol m^−3^, yielding σ_i_ = 1.732. The mixing efficiency was then defined as $\left(\frac{\sigma_{i}-\sigma }{\sigma_{i}}\right)\times 100\left(\%\right)$.

### 4.5 LNP Composition and Synthesis

The organic (lipid) phase consisted of SM‐102, DSPC, cholesterol, and DMG‐PEG2000 (50:10:38.5:1.5 mol%) dissolved in ethanol at a total lipid concentration of 3.125 mm. The aqueous phase contained the indicated nucleic acids dissolved in 100 mm citrate buffer (pH 4), and formulations were prepared at an N/P ratio of 6. For LNP production using the 3D‐printed mixing chamber, the organic and aqueous phases were injected at 5 and 15 mL min^−1^, respectively. For bulk mixing, 2.25 mL of lipid solution was added to 6.75 mL of the eGFP mRNA solution using a syringe, followed by vortex mixing at 3200 rpm for 5 s. The resulting dispersion was then transferred to a syringe and passed through an extrusion filter at 20 mL min^−1^. For the 3DM condition, samples were collected 3 s after the onset of outflow, while those of 3DM‐F condition were collected after 10 s, once the LNP pre‐mix began to exit the system. As a control, mRNA‐LNPs were formulated using the commercial microfluidic mixer (Ignite, NanoAssemblr, Cytiva) under the same 3:1 flow‐rate ratio but at a lower total flow rate of 12 mL min^−1^, corresponding to the manufacturer‐recommended operating condition for LNP synthesis. When required, LNP suspensions were dialyzed against DPBS (pH 7.4) and further purified using Amicon Ultra centrifugal filters (50 kDa MWCO) at 6000 rpm for three cycles to remove residual ethanol.

### 4.6 LNP Characterization

The Z‐average diameter, polydispersity index (PDI), and zeta potential of all LNP formulations were measured using a Zetasizer Nano ZS (Malvern Panalytical). Each sample was measured in triplicate. Prior to analysis, dialyzed samples were diluted 1:100 in DPBS (10 µL sample in 990 µL DPBS), whereas non‐dialyzed samples were diluted 1:10 (100 µL sample in 900 µL DPBS). mRNA encapsulation efficiency was determined using the Quant‐iT RiboGreen RNA Assay Kit. LNPs were diluted either in TE buffer (10 mM Tris‐HCl, 1 mm EDTA, pH 7.5) to quantify unencapsulated (free) mRNA or in 0.5% Triton X‐100 in TE buffer to lyse LNPs and quantify total mRNA. RiboGreen reagent was added to samples and RNA standards, followed by incubation for 10 min at room temperature in the dark. Fluorescence was measured at excitation/emission wavelengths of 485/520 nm using a Hidex microplate reader. Encapsulation efficiency (%) was calculated as $\frac{m_{\mathrm{total}}-m_{\mathrm{free}}}{m_{\mathrm{total}}}*100\left(\%\right)$, where *m*_total_ is the total mRNA measured after lysis, and *m*_free_ is the unencapsulated mRNA measured without lysis. Process yield (%) was calculated as $\frac{m_{\mathrm{total}}-m_{\mathrm{free}}}{m_{\mathrm{input}}}*100\left(\%\right)$, where *m*_input_ is the initial amount of mRNA used for formulation.

### 4.7 Cell Culture

The fibroblast cell line NIH‐3T3 was cultured in DMEM supplemented with 10% v/v FBS and 1% v/v Pen Strep. Cells were maintained at 37°C in a humidified incubator with 5% CO_2_.

### 4.8 In Vitro Validation of mRNA‐Loaded LNPs

NIH‐3T3 cells were used to evaluate mRNA‐LNP formulations in vitro. For cytotoxicity, cellular association, and protein expression studies, LNPs were applied at a final concentration of 0.1 µg µL^−1^. Cells were seeded at 1 × 10^5^ cells per well in 96‐well microplates and cultured overnight to facilitate attachment. Subsequently, cells were treated with LNPs containing 1 µg of mRNA, while DPBS served as a negative control. For cytotoxicity measurements, cells were washed three times with DPBS at 24 and 48 h post‐treatment, followed by the addition of CCK‐8 reagent (10 µL per well) and incubation for 4 h. Absorbance at 450 nm was measured using a Hidex microplate reader, and relative viability was calculated by normalizing to the DPBS control. To qualitatively visualize cell viability, a live/dead assay was performed by incubating the cells with a working solution of 1 µm calcein acetoxymethylester and 2 µg mL^−1^ propidium iodide, followed by observation via fluorescence microscopy. Time‐dependent cellular association was assessed using DiD‐labeled LNPs. To avoid spectral overlap with eGFP expression, LNPs were labeled with DiD to a final dye concentration of 10 µM prior to treatment. At 1, 2, and 4 h after dosing, cells were washed three times with DPBS to remove non‐associated LNPs. DiD fluorescence was quantified at excitation/emission wavelengths of 640/665 nm, and cellular association was reported as the percentage of retained DiD signal relative to the initially applied dose. Protein expression was evaluated by fluorescence microscopy and flow cytometry. After 24 h of treatment, cells were washed three times with DPBS and detached with trypsin. Cellular uptake and eGFP expression were quantified using a CytoFLEX S (Beckman Coulter) and visualized using fluorescence microscopy.

## Conflicts of Interest

Leekang Jeon, Gi‐Ra Yi, and Hyomin Lee are inventors on a patent application related to the 3D‐printed milli‐fluidic hybrid mixing–extrusion platform described in this manuscript, and the associated intellectual property is being pursued for commercialization. The remaining authors declare no conflict of interest.

## Supporting information




**Supporting File 1**: smll74386‐sup‐0001‐SuppMat.pdf.

**Supporting File 2**: smll74386‐sup‐0002‐MovieS1.mp4.

**Supporting File 3**: smll74386‐sup‐0003‐MovieS2.mp4.

## Data Availability

The data that support the findings of this study are available in the supplementary material of this article.
