# Supplementary material for: 3D‐Printed Milli‐Fluidic Mixing‐Extrusion Platform for Continuous, High‐Throughput LNP Production
Source: Small. 2026 Jul 3;22(48):e74386. doi: 10.1002/smll.74386 (PMC13509044; doi:10.1002/smll.74386)
Supplement: Supplementary file 1 [file SMLL-22-e74386-s003.pdf]

Supporting Information

3D-Printed Milli-Fluidic Mixing-Extrusion Platform for Continuous, High-Throughput LNP Production

Leekang Jeon†, Seeun Kim†, Hanjin Seo†, Hyun Su Lee, Gi-Su Na, Gi-Ra Yi, Hyomin Lee**

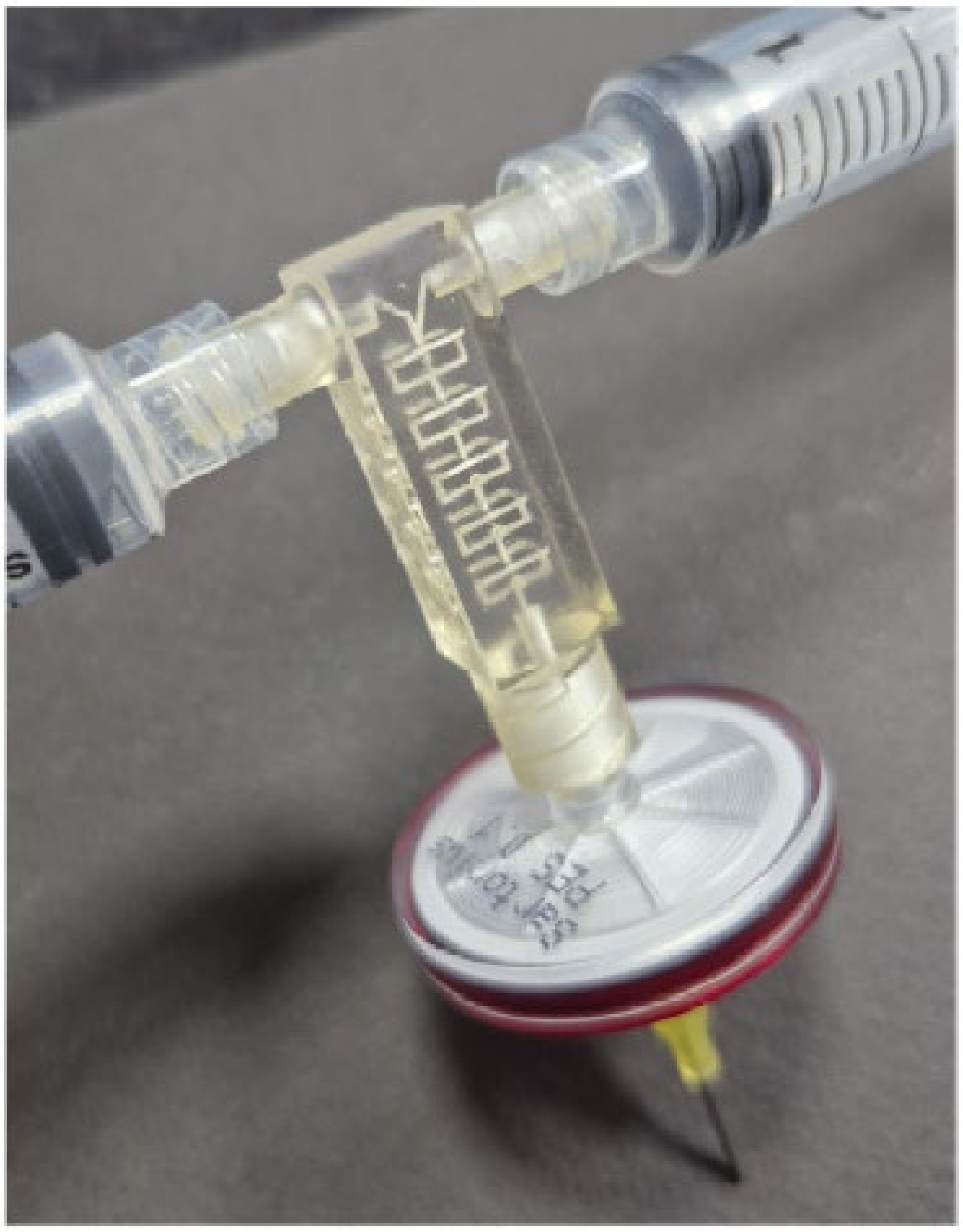

Figure S1. Photograph showing the assembly of the 3D-printed mixing chamber with inline extrusion filter and syringes. The 3D-printed mixer is equipped with Luer-lock fittings to enable direct connection to syringes and the filter module. A polyethersulfone syringe filter (PES, 0.1 μm pore size) was used as the inline extrusion filter.

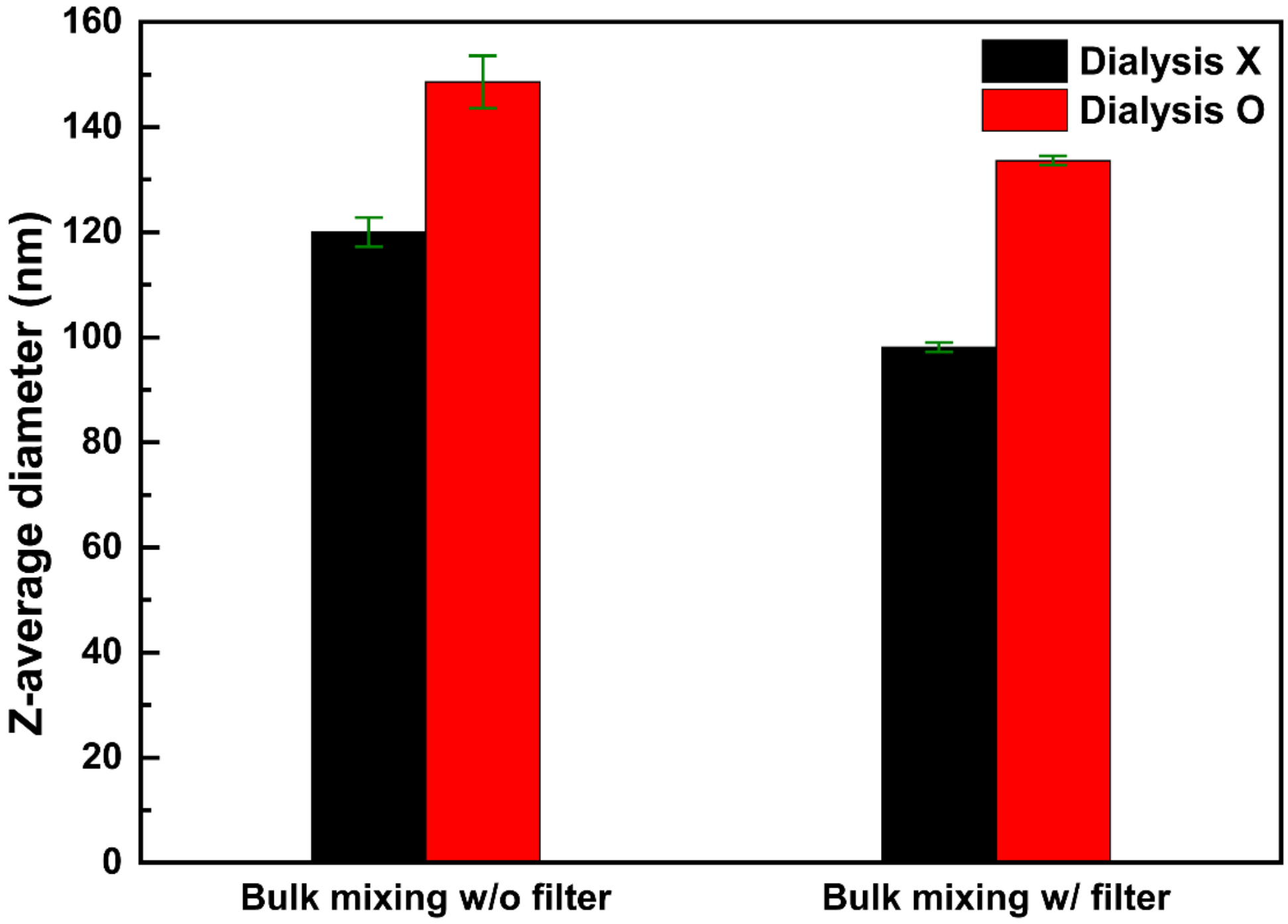

	Bulk mixing w/o filter	Bulk mixing w/ filter
Dialysis X	120.03 nm (0.092)	98.09 nm (0.008)
Dialysis O	148.57 nm (0.121)	133.63 nm (0.083)

Figure S2. Effect of extrusion filter on LNP size for the bulk-mixing workflow. Although incorporation of the filter reduced particle diameter, LNPs produced by bulk mixing remained larger than those generated using the 3D-printed mixers. Error bars represent standard deviation (SD) in which values are reported as mean ± SD (n = 3). Data in the table represent average diameter and polydispersity index (PDI).

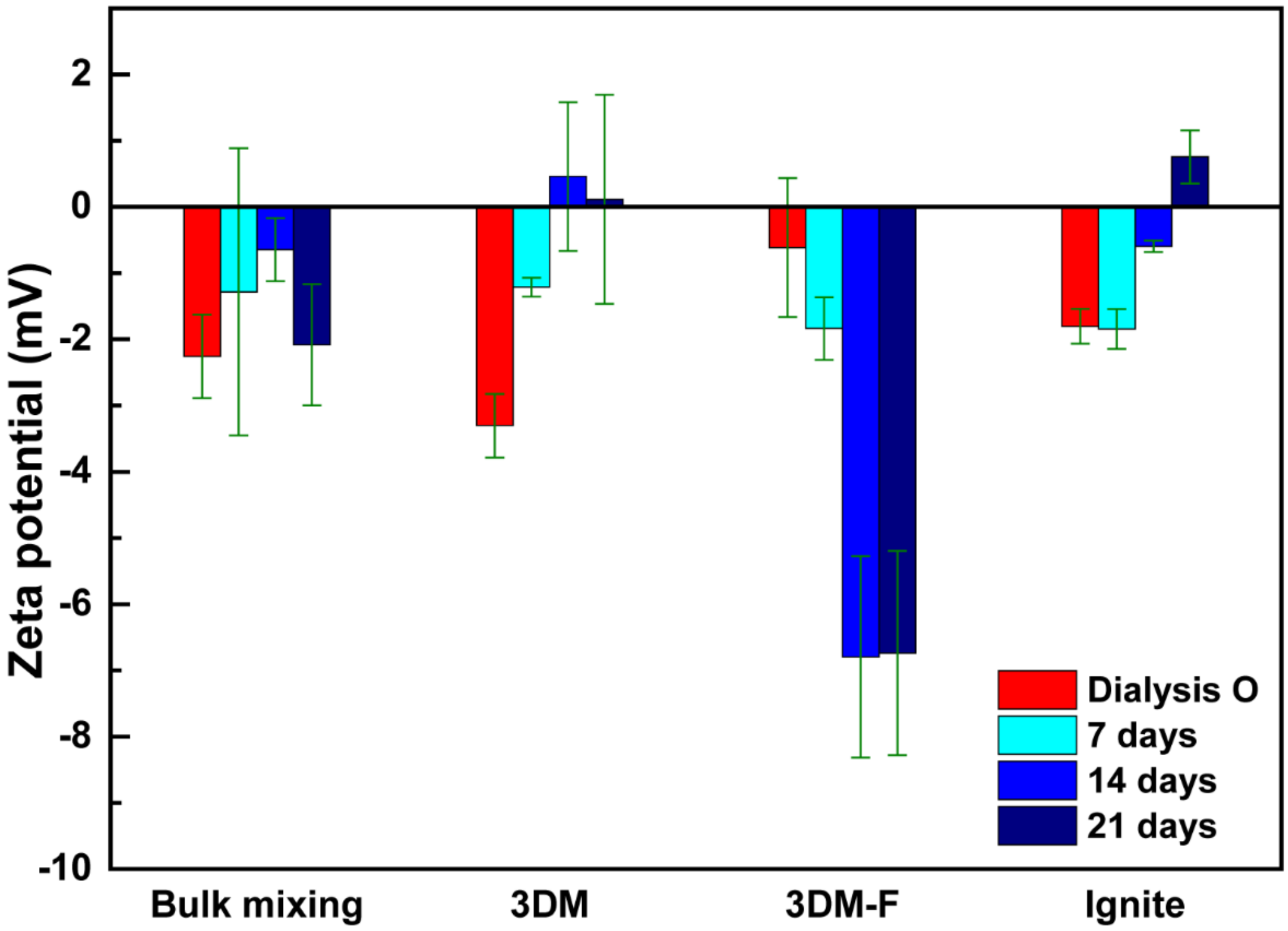

Figure S3. Zeta potential of LNPs produced using four different synthesis workflows. All formulations exhibited near-neutral surface charge, with zeta potentials remaining within −10 to +10 mV across measurements. Error bars represent standard deviation (SD) in which values are reported as mean ± SD (n = 3).

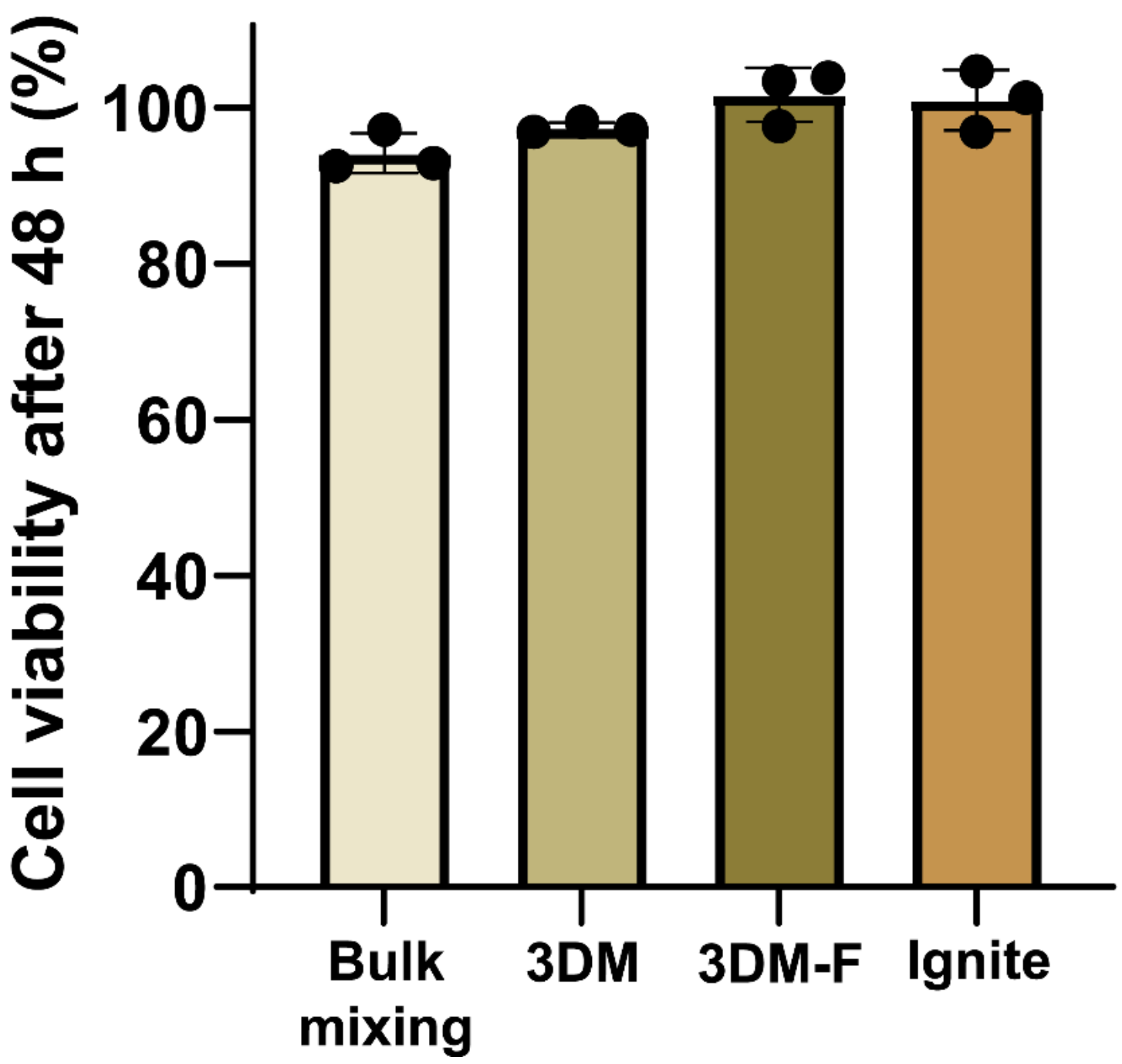

Figure S4. Plot showing cell viability after 48 h quantified using the CCK-8 assay.

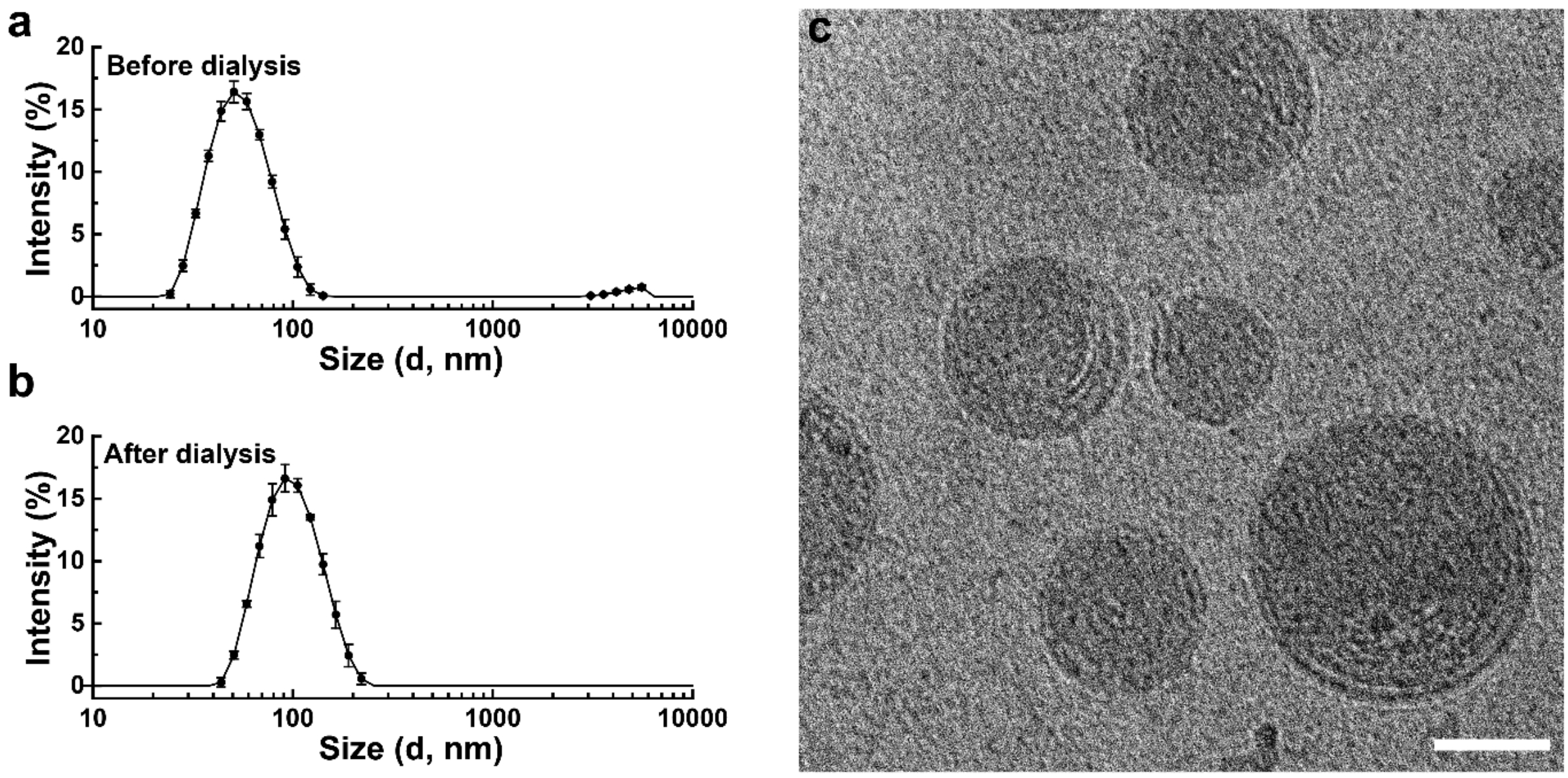

Figure S5. Size distributions of the LNP dispersion generated after 30 min of continuous operation (a) before and (b) after dialysis. (c) Cryo-TEM image of the stabilized LNPs obtained after 30 min of operation followed by dialysis. Scale bar represents 50 nm.

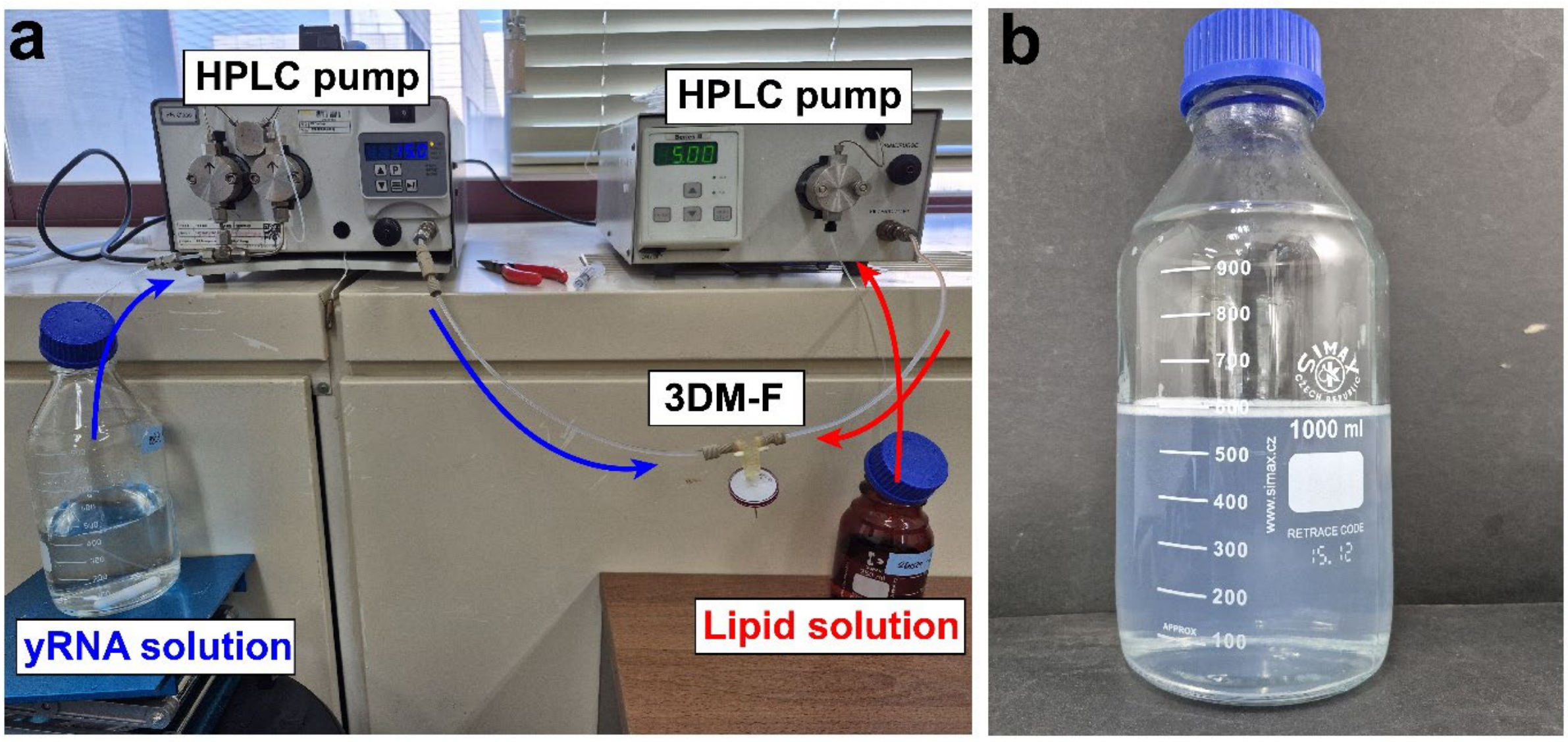

Figure S6. (a) Configuration of the continuous LNP synthesis system using an HPLC pump and (b) the LNP dispersion sample collected during the 30-min synthesis run.

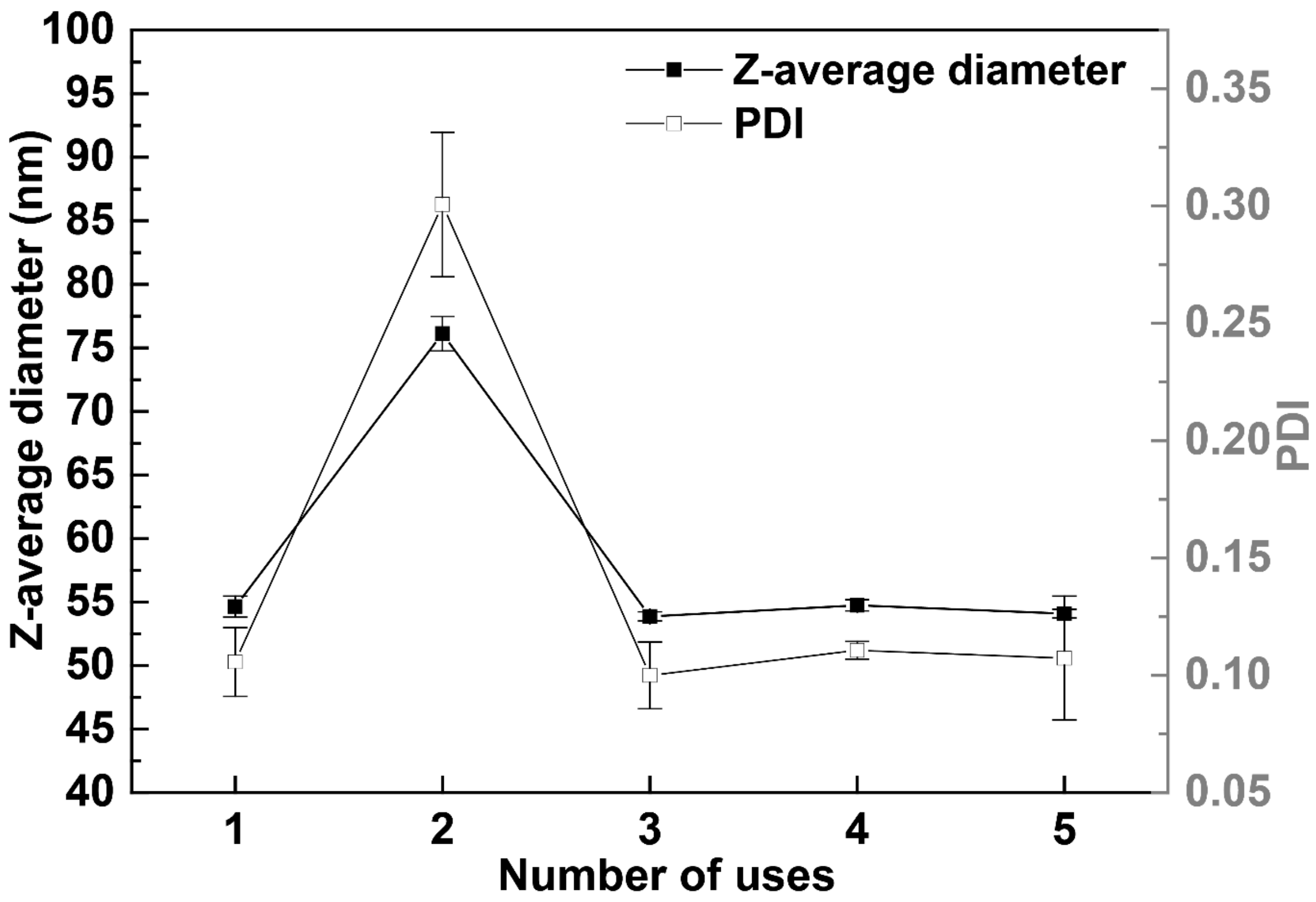

Figure S7. Z-average diameter and PDI of LNPs (prior to dialysis) as a function of the number of reuse cycles using 3DM-F, where each cycle includes 30 s of operation followed by sequential washing with water, ethanol, and PBS.

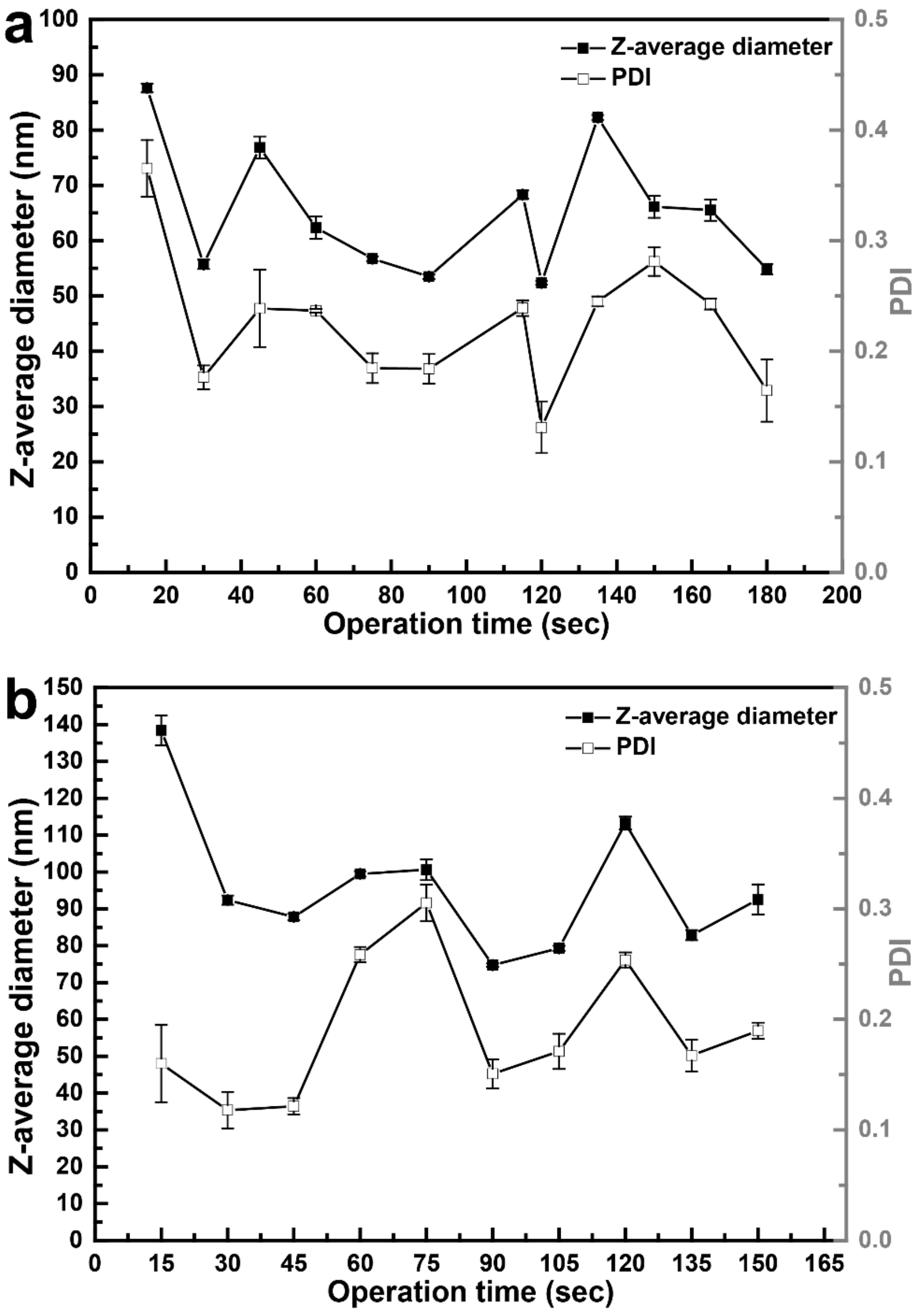

Figure S8. LNP size stability during a 3 min synthesis run at a flow rate of 20 mL min^{-1} using either (a) a polyethersulfone (PES) inline filter or (b) a polytetrafluoroethylene (PTFE) inline filter, each with a pore size of 100 nm. LNPs produced with a PTFE filter exhibited larger diameters than those prepared using a PES filter.

Supporting Movies

Movie S1. LNP synthesis using a 3D mixing chamber with inline extrusion filter.

Movie S2. Visualization of water–ethanol mixing simulations in 1D, 2D, and 3D mixers.
